# Cutting Risk, Not Just Skin—An International Survey on the Role of Preoperative Lab Values in Risk Stratification for Plastic and Reconstructive Surgery

**DOI:** 10.3390/jcm14217686

**Published:** 2025-10-29

**Authors:** Michael Alfertshofer, Joanna Kempa-Timler, Nicholas Moellhoff, Samuel Knoedler, Sinan Mert, Leonard Knoedler, Hans-Günther Machens, P. Niclas Broer, Robin Hartmann, Anna Kasielska-Trojan, Max Heiland, Steffen Koerdt, Philipp Moog

**Affiliations:** 1 Department of Plastic and Hand Surgery, Klinikum Rechts der Isar, Technical University Munich, 81675 Munich, Germany; 2 Department of Oral and Maxillofacial Surgery, Charité—Universitätsmedizin Berlin, Corporate Member of Freie Universität Berlin and Humboldt-Universität zu Berlin, 13353 Berlin, Germany; 3 Plastic, Reconstructive and Aesthetic Surgery Clinic, Institute of Surgery, Medical University of Lodz, 90-151 Lodz, Poland; 4 Division of Hand, Plastic and Aesthetic Surgery, Ludwig-Maximilians-University Munich, 80336 Munich, Germany; 5 Department of Plastic, Hand and Burn Surgery, Klinikum Bogenhausen, Technical University Munich, 81675 Munich, Germany; 6 Department of Oral and Maxillofacial Surgery, Ludwig-Maximilians-University Munich, 80336 Munich, Germany

**Keywords:** preoperative risk stratification, lab values, risk assessment, postoperative complications

## Abstract

**Background/Objectives:** Plastic and reconstructive surgery (PRS) is characterized by its wide range of techniques and procedures, aiming to address the need for individualized treatment approaches. As PRS is predominantly performed in an elective and non-emergency setting, a thorough preoperative risk assessment through standardized diagnostics remains essential. Lab testing is often routinely performed, yet its overall role and how specific parameters are currently used to stratify risk in PRS is poorly understood. We herein aim to explore the role of preoperative lab value testing and characterize current practices, perceived importance, and variability in their use for risk stratification. **Methods:** We conducted an anonymous, web-based cross-sectional survey of international PRS surgeons. Survey items captured demographics, routine preoperative assessment methods, ordering frequency of laboratory tests, and their perceived importance. Group comparisons were stratified by work setting, years of experience, and PRS subspecialization. **Results:** A total of 140 PRS surgeons from 24 countries completed the survey. Clinical evaluation (97.9%) and laboratory testing (84.3%) were the most common risk assessment methods investigated in our study; 70.7% agreed that preoperative lab values are important for surgical risk stratification while 64.3% would adopt a standardized lab-based risk assessment tool. The most ordered lab tests were hemoglobin (80.0%), hematocrit (76.4%), platelets (69.3%), creatinine (68.6%), and white blood cell count (67.1%). Hospital-based PRS surgeons ordered creatinine, WBC, INR and albumin more often and rated albumin of greater importance compared with PRS surgeons based in private practice. **Conclusions:** Preoperative labs are widely used in PRS with emphasis on hematologic and coagulation parameters, in both hospitals and private practices. Broad consensus on the importance of preoperative lab values in surgical risk stratification and a willingness to adopt a standardized, lab-based risk assessment tool highlight the need to harmonize current practice and integrate specific labs into standardized procedure-specific risk stratification.

## 1. Introduction

Plastic and reconstructive surgery (PRS) comprises a wide span of elective aesthetic procedures, complex oncologic and traumatologic reconstructions, hand surgery, as well as burn care, each with distinct physiologic demands and complication profiles [1,2]. Considering the diversity of surgical indications and patient populations, PRS surgeons must not only possess technical expertise but also a comprehensive understanding of (patho-)physiology and systemic patient health [3,4]. Standardized and effective preoperative risk assessments are therefore essential to ensuring patient safety and optimizing surgical outcomes. While clinical history and examination remain essential, lab testing is ubiquitously employed in routine preoperative diagnostics as it offers an objective real-time snapshot of organ function and homeostasis immediately relevant to perioperative decision-making [5,6,7].

Specific lab values cater directly and indirectly to PRS-specific risks and complications; for instance, hematocrit (HCT) and platelet/coagulation assays inform bleeding risk and likeliness of hematoma formation, thereby being of special relevance in microvascular cases for flap perfusion [8,9,10]. Creatinine and related renal markers may influence anesthetic choice, fluid and electrolyte balance, and drug dosing [11,12] while glucose levels can inform infection risk and wound-healing complications [13,14,15], and albumin reflects nutritional reserve and hemostatic balance [16,17]. However, despite their recognized physiological relevance and routine availability, there is striking heterogeneity on the selection of tests and when they are ordered [18,19]. This variability likely arises from differences in hospital-specific case mix (e.g., aesthetic vs. oncologic reconstruction), practice setting (hospital vs. private practice), local order sets and cost structures, and, lastly, the absence of PRS-specific guidelines and decision pathways linking lab value results to perioperative actions.

Previous risk assessment models in PRS have focused on patient comorbidities or operative complexity. The role of standardized diagnostics, especially routine preoperative laboratory testing, and how they are leveraged in daily clinical practice remains largely untapped. To clarify current practice and define needs for standardization, we conducted an international survey of PRS surgeons across subspecialties and work settings. Our objectives were to characterize the frequency with which individual laboratory tests are ordered, evaluate how important surgeons perceive these tests for preoperative risk stratification, and identify patterns of variation that help to provide an evidence-based foundation for a standardized and procedure-specific lab framework aiming to enhance patient safety in PRS.

## 2. Material and Methods

### 2.1. Study Design

This cross-sectional study was designed as an international, web-based, anonymous survey targeting in-training and board-certified PRS surgeons. The survey aimed to analyze current practices and perceptions on the use of preoperative lab values in risk stratification in PRS cases. Questions were developed by the study authors and were subsequently reviewed by a panel of three external board-certified PRS surgeons with expertise in different subspecializations (i.e., aesthetic, reconstructive, and hand surgery) to ensure validity, clarity, and relevance. Prior to survey distribution, changes were then incorporated in the final version according to their feedback. The final survey included items on demographics (i.e., age, gender, geographic region, years of being a board-certified PRS surgeon, work institution, and primary subspecialty focus), as well as on the routine practice, perceived importance, and clinical implications of specific preoperative laboratory parameters with optional free-text questions (Supplementary Table S1). Consistent with the set analyzed in the American College of Surgeons (ACS) National Surgical Quality Improvement Program (NSQIP) database, the following lab values were included in the survey: hemoglobin (Hb), hematocrit (HCT), platelet count, white blood cell count (WBC), creatinine, blood urea nitrogen (BUN), albumin, blood glucose, bilirubin, alkaline phosphatase (AP), glutamate oxaloacetate transaminase (GOT), prothrombin time (PT), international normalized ratio (INR), partial thromboplastin time (PTT), and sodium, while we additionally included C-reactive protein (CRP) and potassium [20].

Responses were collected using Google Forms (Google LLC, Mountain View, CA, USA) and LimeSurvey (LimeSurvey GmbH, Hamburg, Germany). At all times, study participation was voluntary, and no personal identifiers were collected with all responses being anonymous. Informed consent was implied by submission of the completed survey, as stated in the participant information. The study protocol was reviewed under §15 of the Bavarian professional code by the Ethics Commission of the Technical University of Munich and ethics approval was obtained under IRB number 2024-82-S-CB.

### 2.2. Participant Recruitment

To ensure broad international representation, potential participants were identified through random sampling of membership lists of national and international plastic surgery societies. Email addresses were sourced from these publicly accessible directories. Moreover, the survey was distributed via the following societies’ newsletters to their respective members: the Australian Society of Plastic Surgeons (ASPS), the German Society of Plastic, Reconstructive and Aesthetic Surgery (DGPRÄC) and the Swiss Association of Plastic Reconstructive and Aesthetic Surgeons (SGPRAC). A reminder email was sent at 2 weeks to increase participation rate with the survey open for a total period of 16 weeks. Only completed surveys were included in the final analysis. As recruitment occurred through multiple overlapping channels (i.e., society newsletters, public society directory mails, ad-hoc forwarding) the total number of PRS surgeons reached was unknown, and a reliable response rate could not be calculated.

### 2.3. Data Handling and Statistical Analysis

Survey responses were collected and exported in an excel spreadsheet for analysis. Continuous variables are reported as mean ± standard deviation, and categorical variables as counts and percentages. Group comparisons for categorical variables used the χ^2^ test or Fisher’s exact test when expected cell counts were small. For continuous and ordinal (Likert-type) measures, between-group comparisons used the Mann–Whitney U test (two groups) or the Kruskal–Wallis test (≥3 groups). Likert-scale importance ratings were treated as approximately interval to facilitate comparability across items and are summarized as means ± standard deviations to allow for better readability. The correlation between ordering frequency and perceived importance was assessed using two-sided Spearman’s rho. All analyses were performed using SPSS Statistics 27 (IBM Corp., Armonk, NY, USA) with a two-tailed *p*-value <0.05 considered statistically significant to guide conclusions.

## 3. Results

### 3.1. Participant Demographic Data

**Figure 1:**
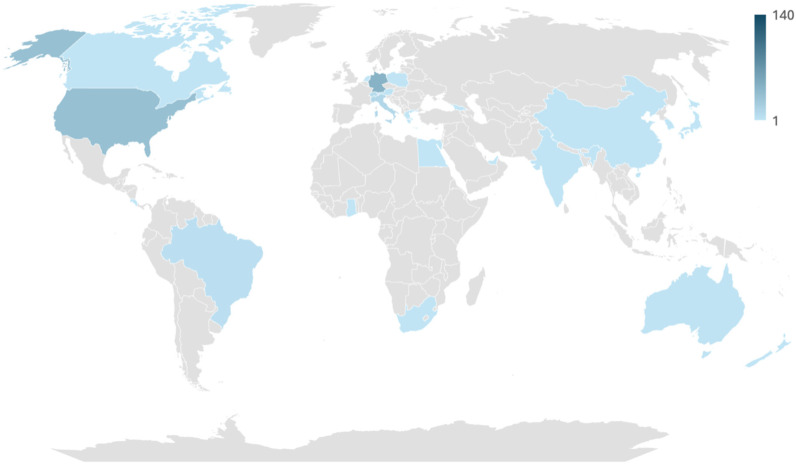
Choropleth world map visualizing the global distribution of survey respondents. Darker shading indicates more respondents; countries without respondents are shown in gray.

**Figure 2:**
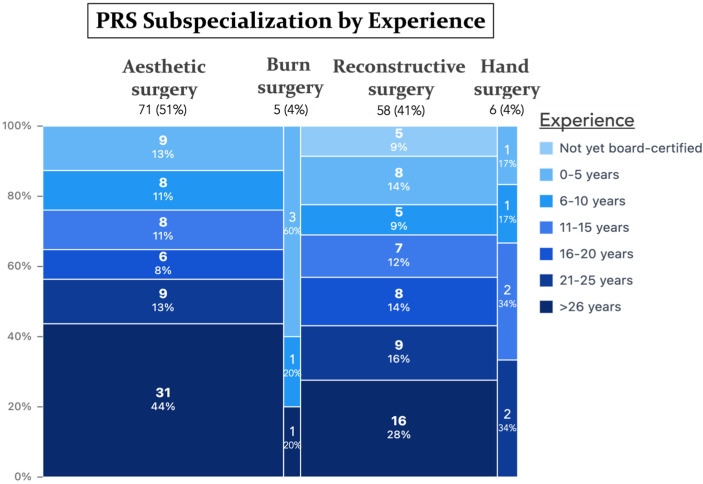
Marimekko chart showing the distribution of PRS subspecializations stratified by years of professional experience. Each rectangle’s width represents the relative proportion of each PRS subspecialization, while its height reflects the work experience as a PRS surgeon in years within that respective subspecialty.

**Figure 3:**
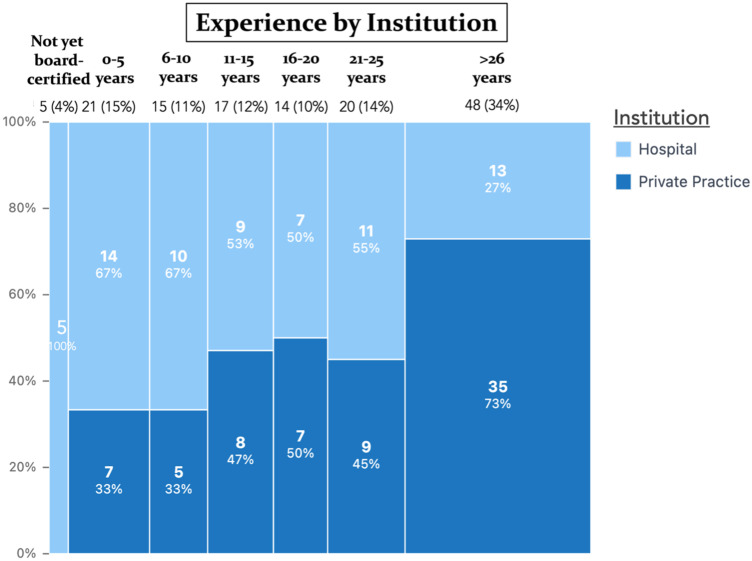
Marimekko chart showing the distribution of work experience levels among doctors working in hospital vs. private practice. Each rectangle’s width represents the relative proportion of work experience as a PRS surgeon in years, while its height reflects the percentage of respondents working in hospitals versus private practices within that experience group.

**Figure 4:**
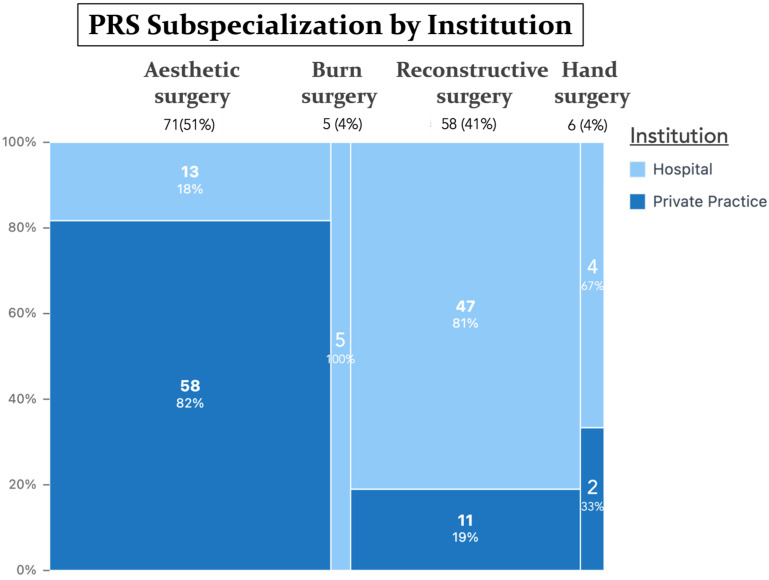
Marimekko chart showing the distribution of PRS subspecializations among doctors working in hospital vs. private practice. Each rectangle’s width represents the relative proportion of each PRS subspecialization, while its height reflects the percentage of respondents working in hospitals versus private practices within that respective subspecialty.

A total of 140 plastic surgeons completed the survey (103 males, 37 females), with a mean age of 52.3 ± 14.2 years [range 25–85]. Participants represented 24 countries, with Germany being the most frequent (32.9%) (Figure 1). Over one-third (34.3%) had been board-certified for ≥26 years. Practice settings were evenly distributed between hospitals (49.3%) and private practices (50.7%). The main surgical focuses were aesthetic (50.7%), reconstructive (41.4%), hand (4.3%), and burn surgery (3.6%). A complete summary of demographic information is shown in Table 1 and Figure 2, Figure 3 and Figure 4.

### 3.2. Preoperative Risk Assessment Practices

Nearly all surgeons (97.9%) reported routinely conducting a clinical evaluation, followed by laboratory tests (84.3%), imaging (73.6%), and multidisciplinary case discussions (72.1%). Intuition was cited by 60.7%, while standardized risk tools (37.1%) and patient-reported outcomes (34.3%) were least used. Hospital-based surgeons were more likely than private practitioners to include multidisciplinary discussions (79.7% vs. 64.8%, *p* = 0.049) and imaging studies (81.2% vs. 66.2%, *p* = 0.045). Across subspecialties, reconstructive surgeons most frequently employed such discussions (84.5%), compared with hand (66.7%), aesthetic (64.8%), and burn surgery (40.0%) (*p* = 0.030).

### 3.3. Preoperative Laboratory Testing

Most participants (70.7%) agreed that routine lab values are important for risk stratification, and 64.3% reported they would likely adopt a standardized lab-based risk tool. The most ordered preoperative lab tests were Hb (80.0%), HCT (76.4%), platelets (69.3%), creatinine (68.6%), WBC (67.1%), sodium (66.4%), PTT (63.6%), potassium (60.7%), INR (57.9%), blood glucose (51.4%), and PT (46.4%). Less frequently ordered were CRP (41.4%), GOT (37.1%), BUN (36.4%), albumin (35.7%), bilirubin (31.4%), and AP (28.6%).

**Figure 5:**
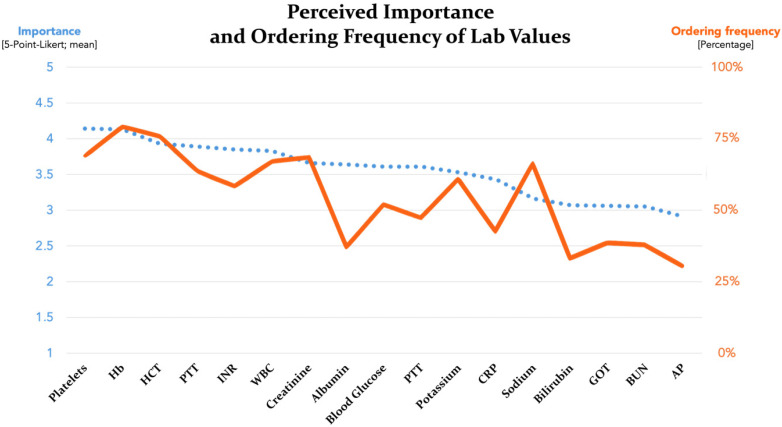
Line graphs showing the perceived importance of lab values for predicting postoperative complications, shown in descending order (blue dotted line: mean value of 5-point Likert-scale ratings; orange line: ordering frequency in %).

The perceived importance of these parameters largely mirrored ordering frequency: highest for platelets (4.14), hemoglobin (4.13), hematocrit (3.93), PTT (3.89), INR (3.85), WBC (3.83), creatinine (3.66), albumin (3.64), blood glucose (3.61), PT (3.61), potassium (3.53), and CRP (3.43) and lowest for sodium (3.16), bilirubin (3.07), GOT (3.06), BUN (3.05), and AP (2.91). A strong correlation was found between ordering frequency and perceived importance for all values (*p* < 0.001). The complete data are summarized in Figure 5 and Table 2.

### 3.4. Differences by Practice Setting and PRS Subspecialization

Hospital-based PRS surgeons ordered creatinine (76.8% vs. 60.6%; *p* = 0.038) albumin (46.4% vs. 25.4%; *p* = 0.009), WBC (76.8% vs. 57.7%; *p* = 0.016), and INR (68.1% vs. 47.9%; *p* = 0.015) more often on a statistically significant level when compared with private-practice-based PRS surgeons. Subspecialty comparisons showed creatinine testing was universal in burn surgery (100%) but less common in aesthetic (66.2%), hand (16.7%), and reconstructive surgery (74.1%), with *p* = 0.013. INR was ordered most frequently in burn (100%) and reconstructive surgery (69.0%) versus aesthetic (46.5%) and hand (50.0%), with *p* = 0.015. Hemoglobin was prioritized in aesthetic (81.7%) and reconstructive (84.5%), when compared with burn (60.0%) and hand surgery (33.3%), with *p* = 0.016.

Perceived importance ratings also varied: albumin was rated highest in burn (4.60) and lowest in aesthetic surgery (3.39), with *p* = 0.025; PTT highest in reconstructive (4.26) vs. aesthetic (3.58), with *p* = 0.025; and INR highest in reconstructive and hand surgery (4.17) vs. aesthetic (3.55), with *p* = 0.036. Hospital-based surgeons rated albumin to be more important in preoperative risk stratification than private practitioners (3.91 vs. 3.40, *p* = 0.012).

Responses to free-text questions are summarized in Supplementary Table S1.

## 4. Discussion

In this international survey of 140 PRS respondents from 24 countries, we aimed to provide a contemporary overview of how international PRS surgeons incorporate routine preoperative lab testing into their overall risk assessment. It was identified that preoperative lab values are considered a core element in preoperative risk stratification for 84.3% of respondents, second only to clinical examination, which was named by almost all respondents (97.9%). The majority of respondents expressed willingness to adopt a validated, data-driven risk assessment tool based on preoperative lab values to aid their risk assessment strategies. These results suggest that variability in current practice stems less from general skepticism toward the role of lab values in preoperative risk assessment and more from the lack of an easy-to-use, evidence-based instrument tailored to PRS.

Across work settings and subspecialties, PRS surgeons prioritized hematologic and coagulation parameters (i.e., Hb, HCT, platelets, PTT/INR) and creatinine. This aligns with perioperative concerns central to PRS: bleeding/hematoma, oxygen delivery and flap perfusion, coagulation and wound healing as well as dosing of anesthetic medication in renal impairment [21].

Interestingly, lab values reflecting liver function and status such as bilirubin, AP, GOT and BUN, were consistently rated lower in both ordering frequency and perceived importance. Several factors likely underpin this pattern: In PRS, especially outpatient aesthetic procedures, comparatively healthy patients with a low prevalence of clinically meaningful liver diseases are seen and mild, isolated transaminase or cholestatic abnormalities rarely would alter perioperative strategies in PRS [22]. Further, bleeding risk and anesthetic safety are more directly captured by coagulation assays (INR/PTT), platelet count and creatinine. Hence, PRS surgeons often rely on history and a medication review with anesthesiology screening to eventually trigger hepatobiliary work-ups when indicated rather than routinely ordering such lab values.

Our data demonstrate a strong correlation between ordering frequency and perceived importance of lab values for surgical risk assessment. Interestingly, two lab values displayed a noteworthy mismatch between both variables: albumin and sodium. Although albumin was rated moderately to highly important (rank 8), it was among the least commonly ordered tests overall (rank 15). Sodium was ordered frequently (rank 6) despite low perceived importance (rank 13) for surgical risk assessment. Several mechanisms may explain this discrepancy: (i) default lab value bundles embedded in institutional order sets, often including electrolyte workup (e.g., potassium and sodium), while sparing nutritional markers (e.g., albumin) [23]; (ii) variable reimbursement and cost visibility [24,25,26]; and (iii) absence of guidelines and clinical experience on how to act on borderline values (e.g., what threshold of hypoalbuminemia warrants case postponement with nutritional optimization) [27,28]. This proposed “actionability gap” argues for the development of procedure-specific algorithms that relate thresholds with specific decision rules (e.g., case postponement, optimization).

Hospital-based PRS surgeons ordered creatinine, WBC, INR, and albumin significantly more often and valued albumin higher than private-practice-based colleagues. This finding can be explained by the hospital patient cohort, which is often characterized by complex oncologic or traumatologic cases requiring reconstructive surgery, in contrast to the predominantly elective aesthetic procedures encountered in private practice. Patients in the former cohort often warrant a more in-depth preoperative workup, extending beyond clinical examination to include lab values, imaging, and multidisciplinary discussion. This is also mirrored in our data, where hospital-based reconstructive surgeons rely more heavily on multidisciplinary consultations and imaging studies compared with their private-practice-based and non-reconstructive-subspecialist colleagues (i.e., hand, burn, aesthetic surgery). Subspecialty differences were equally intuitive: creatinine and albumin were emphasized by burn surgeons, mirroring volume loss and catabolic stress associated with burns [29,30]. Reconstructive surgeons rated coagulation parameters (PTT, INR) highly, potentially given microvascular considerations [31], and aesthetic surgeons, typically in healthier cohorts, showed lower importance ratings and reported selective rather than routine testing, especially for hemoglobin [32]. These gradients support the notion that a single “universal” preoperative panel is likely inefficient, calling for a subspecialty- and procedure-specific approach to reflect on the specific challenges and complications encountered in the respective field of PRS.

In our study, we included free-text questions to capture more nuanced insights into respondents’ perspectives on the role of lab values in PRS. Among the heterogeneity of responses, three common themes emerged and were particularly noteworthy. First, nutrition and inflammation (i.e., (pre-)albumin, CRP and procalcitonin) were repeatedly named as important variables that deserve research and possibly integration into diagnostic workups. Respondents described improved outcomes after nutritional optimization in cases with hypoalbuminemia, while others underlined the role of CRP or glycated hemoglobin (HbA1c) when infection risk or glycemic control were concerns.

Second, some surgeons expressed interest in AI-powered risk models that adapt to local populations and procedures. In terms of PRS, which is characterized by a wide array of procedures, patient goals, and tissues, machine learning could help translate multivariate lab patterns together with other risk determinants into individualized probabilities of specific complications.

Third, multiple respondents noted that they selectively rather than routinely test healthy aesthetic patients, in line with guidelines that discourage blanket testing in low-risk populations [33,34]. Hence, based on our results, we support a dual imperative: curb non-actionable testing in low-risk pathways, while improving targeted testing in patient cases where optimal preoperative coagulation, organ function and nutritional status is detrimental for surgical success (e.g., anticipated major blood loss, microvascular reconstruction, catabolic states, diabetes, renal or hepatic compromise). Defining thresholds and optimization recommendations will then translate testing into action.

Based on the strong willingness to adopt a lab-value-based risk tool in PRS paired with the “actionability gap” identified in our study (e.g., for albumin) that was identified in our study population, an implementation window opens. We envision a concise, procedure-specific PRS risk tool organized in three layers for non-trivial surgical cases: (1) universal core (Hb/HCT, platelets, WBC, creatinine, coagulation assay), (2) condition-triggered tests (e.g., HbA1c for diabetes, CRP when infection suspected), and (3) risk- or procedure-specific add-ons (e.g., albumin for bariatric or oncologic cases; electrolytes in burn or fluid-shift).

This study, however, is not without limitations. First, as a self-reported survey, our data reflect perceptions rather than audited behavior and are vulnerable to recall and social-desirability bias. Second, voluntary participation introduces selection and nonresponse biases that may limit generalizability, especially in an international context. In this respect, it should be noted that people engaged in academia might be more willing to voluntarily participate in survey studies as a kind of reciprocal activity. Third, several subgroup strata, specifically burn and hand surgery, were naturally small, making those comparisons exploratory and underpowered. Finally, given the study’s nature as a survey, we were unable to analyze contextual drivers such as institutional order-set defaults, payer constraints, local test costs, or anesthesiology policies that likely influence ordering patterns.

Future prospective, multicenter studies investigating the relationship between preoperative laboratory strategies and procedure-specific outcomes in PRS are warranted. This would be a chance to create a set of guidelines—with a layered, evidence-based lab value panel with explicit thresholds and subsequent management pathways. Creating this standardized approach could enhance patient safety while minimizing low-value testing. Lastly, the aspect of additional healthcare cost and complication management associated with overtesting versus undertesting warrants a structured investigation to allow guiding conclusions.

## 5. Conclusions

This international survey of PRS surgeons shows that hematologic and coagulation markers, together with creatinine, are most consistently prioritized in routine lab-value based preoperative risk assessment. Respondents also emphasized the importance of laboratory values overall and expressed a strong interest in a dedicated lab value-based risk assessment tool. The perceived importance of specific lab values largely mirrored ordering frequency, with mismatches evident for albumin and sodium, showing high perceived importance but less frequent ordering and vice-versa, respectively. PRS subspecialization and practice setting significantly influenced the selection of lab values, with reconstructive and hospital-based surgeons favoring more comprehensive assessments and aesthetic or private-practice surgeons tending toward selective testing in healthier cohorts. These findings support a layered, procedure-specific approach, and future studies should define actionable thresholds and integrate them into pathways to increase patient safety and reduce low-value testing.

## Figures and Tables

**Table 1:** Demographic data of study participants.

	Subgroup	n = 140	%
Gender	Male	103	73.6
	Female	37	26.4
Age	Mean, SD	52.3 ± 14.2	
Years of being a board-certified plastic surgeon	Not yet board certified	5	3.6
	0–5 years	21	15.0
	6–10 years	15	10.7
	11–15 years	17	12.1
	16–20 years	14	10
	21–25 years	20	14.3
	>26 years	48	34.3
Work setting	Hospital (e.g., academic institution)	69	49.3
	Private practice	71	50.7
Primary focus of surgery	Aesthetic surgery	71	50.7
	Reconstructive surgery	58	41.4
	Hand surgery	6	4.3
	Burn surgery	5	3.6

**Table 2:** Summary of routinely ordered preoperative laboratory values and their perceived importance, in descending order.

Percentage of Study Participants Routinely Ordering the Laboratory Value		Perceived Importance of Laboratory Value for Preoperative Risk Stratification	
Hb	80.0% (112)	Platelets	4.14 (1.06)
HCT	76.4% (107)	Hb	4.13 (1.08)
Platelets	69.3% (97)	HCT	3.93 (1.04)
Creatinine	68.6% (96)	PTT	3.89 (1.13)
WBC	67.1% (94)	INR	3.85 (1.16)
Sodium	66.4% (93)	WBC	3.83 (1.04)
PTT	63.6% (89)	Creatinine	3.66 (1.09)
Potassium	60.7% (85)	Albumin	3.64 (1.09)
INR	57.9% (81)	Blood glucose	3.61 (1.14)
Blood glucose	51.4% (72)	PT	3.61 (1.14)
PT	46.4% (65)	Potassium	3.53 (1.08)
CRP	41.4% (58)	CRP	3.43 (1.24)
GOT	37.1% (52)	Sodium	3.16 (1.08)
BUN	36.4% (51)	Bilirubin	3.07 (1.10)
Albumin	35.7% (50)	GOT	3.06 (1.10)
Bilirubin	31.4% (44)	BUN	3.05 (1.04)
AP	28.6% (40)	AP	2.91 (1.08)

## Data Availability

The study data are available from the corresponding author upon reasonable request.

## Supplementary Materials

The following supporting information can be downloaded at https://www.mdpi.com/article/10.3390/jcm14217686/s1, Table S1: Free-text responses from surveyed plastic surgeons regarding how laboratory values influence surgical decision-making, which postoperative complications might have been prevented, in which cases did laboratory results lead to changes in surgical approach, which improvements they would like to see in current risk assessment models in PRS, and if there are specific laboratory tests or markers the survey taker believes to have an impact of preoperative risk stratification in PRS.
