# Supplementary material for: Cutting Risk, Not Just Skin—An International Survey on the Role of Preoperative Lab Values in Risk Stratification for Plastic and Reconstructive Surgery
Source: J Clin Med. 2025 Oct 29;14(21):7686. doi: 10.3390/jcm14217686 (PMC12609558; doi:10.3390/jcm14217686)
Supplement: Supplementary file 1 [file jcm-14-07686-s001.zip › jcm-3944170-supplementary.pdf]

Supplementary Material

Supplementary Table S1: Free-text responses from surveyed plastic surgeons regarding how laboratory values influence surgical decision-making, which postoperative complications might have been prevented, in which cases did laboratory results lead to changes in surgical approach, which improvements they would like to see in current risk assessment models in PRS, and if there are specific laboratory tests or markers the survey taker believes to have an impact of preoperative risk stratification in PRS.

**"Please explain how the above-mentioned values influence your surgical decision-making."**	**"Which postoperative complications have you encountered that you believe could have been predicted or mitigated by a comprehensive assessment of preoperative laboratory values?"**	**"Describe any instances where laboratory values significantly altered your surgical approach or patient management."**	**"What improvements would you like to see in current risk assessment models for PRS?"**	**"Are there specific laboratory tests or markers you believe should be researched further for their predictive value in postoperative complications?"**
I am looking at the coagulation parameters to see how much bleeding I can expect. Also ***CRP*** *allows me to understand if there is an ongoing infection. Also seeing how many* ***platelets*** *and* ***Hb*** *the patient has, is essential to understand the risk of operation.*	*I was doing an abdominoplasty case with extensive muscle movement. This case was done in a patient with impaired renal function. Therefore, kidneys parameter (e.g.* ***creatinine****) is crucial*	*Oftentimes, I cancel surgeries or postpone them when* ***coagulation parameters*** *are pointing towards increased bleeding.*	*I would like to see a risk adjustment based on weight, height and other demographic, patient-individual parameters.*	
Flap survival is highly dependent on ***Hb****!*	*Bleeding could have been controlled better.*	***Platelets*** *are transfused when below a certain threshold (<50k)*		***Albumin****. I have heard it is of great use but currently don't know how to integrate it in my assessment.*
I don't routinely order unless indicated. ***Coagulation*** *for history of excessive bleeding,* ***H/H*** *if history of anemia, glucose, and* ***HbA1C*** *or* ***Fructosamine*** *for diabetic patient.* ***Platelets*** *in patient with low platelets*	*Postoperative bleeding in cases of undiagnosed coagulopathy*			
Determines need for further work up in case of preop anemia or renal impairment as well as identification of coagulopathy prior to surgery involving significant blood loss	*Bleeding risk, thromboembolic complications*	*Anaemic patients, those with abnormal* ***coagulation studies***		
Low ***Hb*** *is a contraindication for some surgery involving high blood loss.*				***Albumin, Protein***
Young healthy adults are mostly my patients. Anesthesia requires only ***Hb*** *for females with no medical problems*	*I'm not sure if only altered laboratory values by itself can cause an eventual complication. It is important to consider the surgery duration time less than six hours, the team expertise, adequate tissue manipulation, early patient mobility, patient motivation to underwent to the procedure indicated and avoid unnecessary use of technology, overall in plastic surgery.*	*Significant alteration of* ***blood glucose****,* ***Hb****,* ***Vitamin D****,* ***Homocystein***	*Use of AI model in which we input all risk factors we know about our patient*	
				Albumin
Big ***electrolyte in-balances*** *contraindicate surgery.*				
	Anemia, renal failure	*Blood transfusion when anemic*		
	Infection parameters*,* ***platelets****,* ***HCT*** *and / or* ***Hb*** *deficits*			
Assessment of ability to heal and avoid infection.	*Bleeding, infection, thrombosis, association with complications / worsening of associated and/or undiagnosed diseases*	*Any alteration is significant in aesthetic surgery and serves as a warning and/or to indicate preventive measures.*		

	Tachyarrythmia and prolonged bleeding.	*We focus on reducing **potassium** levels in patients with elevated potassium to mitigate the risk of cardiovascular incidents. we do this through a staged process with escalating measures: fluid balance, diuretics, potassium-evading medication, etc.*		
	Infection, delayed wound healing, bleeding/hematoma.	*For patients with extremely **low platelets**, **PT**, **aPTT** and long **INR**, i will find a minimum invasive way to treat and maybe prepare some cryoprecipitate before the surgery*		***Electrolytes***
*Low **Hb** calls for transfusion, especially in free-flap cases*		*Often times we transfuse before a flap surgical case*		
	Post operative bleeding			
		*Dismissing of surgery because of low **platelets***	*Artificial intelligence, automated consolidation of different variables*	***Albumin***
*Low **platelets** could lead to dismissing of surgery, infections indicated by **CRP** and **WBC** are relevant other than surgical focus*				
			AI	***Immunonutrition parameters***
*If **hemoglobin** is too low -> no operation like tummy tuck or greater lipos, and **CRP/WBC** is too high and an infection is possible -> no operation, if **glucose**/**HbA1C** is to high -> explaining a higher risk of post operative infections and wound healing disorders*				
*Sometimes we demand further investigation. and even sometimes the surgery is cancelled, if some values (e.g. **INR**) are pathologic in more than 2 controls*				
	Infection, flap perfusion problem, cardio-pulmonary failure, renal failure	*Low **platelets**/much increased platelets: cancellation of operation*	*Risk calculation, helping to decide how to avoid complications, kind of structured risk plan such as: red - yellow - green*	***Vitamin D** levels*
*Low **hemoglobin** and poor cardio-pulmonary function: give blood, **platelets** below 50:000: give platelets etc.*				
Operations are strongly elective. Preoperative routine lab therefore does not apply to most of our patients. Only selective lab tests. In other surroundings lab test very helpful.				
	Wound healing			***Glucose***
Lab values are very important to value the risk before operation				
	Bleeding, promoted wound healing	*Optimizing / supporting insufficient **iron**, **proteins** or others made better outcome*		***Proteins, Iron***
Staging of relative infections or important insufficiencies/ pathological settings				
	Postoperative bleeding, thromboembolic events	*Hypercoagulopathy => switch from free flap to pedicled flap*		
	Bleedings, wound healing issues			
Relative or absolute contraindication	*Wound-healing issues after breast reduction in possibly pre-diabetic patient*	*Low **Hb** = no surgery*		
If it is over 2 times of upper range of normal values or less than half of lover range, i will consider to postpone the selective operation				
In our practice, preop labs are only ordered for patients beyond a certain age. If the values are not within normal limits, we refer them back to their primary care doctor for evaluation.			*A simpler risk assessment that is consistent for everyone*	
For instance, transfusion, and adaption of medication dosing			*Speed and cost*	*Coagulation tests (e.g. fibrinogen, antithrombin) for free flaps*

	Blood transfusion	*Low **Hb**, high **INR***	*Remind plastic surgeons to follow ASA guidelines and stop ordering lab tests on ASA I patients.*	
		*Lower **Hb**, low **PTT** or **INR***		
*As my field is burn surgery, I am always interested in the volume status of my patients. For this, I need **electrolytes** and **albumin**. Also a good indicator for volume loss - especially short term - is the dynamic of the **hematocrite***				
	*Really bad bleeding which could have been predicted by ordering **platelets** and **INR***	*For really big cases, occasionally I cancel the surgery if the risk profile is just too unfavorable for me.*		***Albumin***
				***Mitochondrial function**; **inflammatory markers**; baseline **vitamins and minerals** i.e. functional health of tissues*
				Transferrin
				Pre-albumin
I expect normal values prior to surgery and if there are abnormal values, i consider further investigations				
The basics for choosing preop lab has to remain the clinical evaluation				
*I don't look at labs results as a predictor of post operative complications per se, but rather suitability for surgery/anesthesia. Obviously someone with a **PTT** of 60 would likely lead to post operative bleeding and hematoma, for example.*				
They are done to assess the general condition of the patient or fitness for surgery/anesthesia				
For healthy patients with no history or risk factors we do not routinely order pre-op labs. I do only aesthetic surgery so patient selection is biased towards extremely healthy clientele				
I order preop tests of any sort when and where relevant to my history and examination findings and in conjunction with my anesthetist				
*The influence of impaired **coagulation** directly shows in surgical reality. Also, the influence of **electrolytes** on cardiovascular incidents (tachyarrythmia in elevated potassium) is something I have seen more than once.*	*Hematoma, severe bleeding*	***Hemoglobin**, **platelets** low level*		
				TSH
*Most important for my surgical field are **coagulation parameters** and **hemoglobin**, moreover **MCH**, **MCHC**.*				
Lab values are absolutely critical to my preoperative decision-making, as they provide a comprehensive image of a patient's physiological state and their ability to withstand the stress of surgery.				
			More dynamic, real-time scoring systems that integrate with EMRs, rather than static one-size-fits-all calculators.	
			The approach must be tailored to the situation	
I think they are essential to management intra- and postoperatively				
If bacterial or viral infection is detected in a routine blood test, there is a 90% chance that patients will experience delayed wound healing, wound dehiscence, and infection in the postoperative period.	*Lab work specially important in postbariatric patients: **Nutrition values, e.g. albumin** and **coagulation factors** etc. or in polymorbid pat. population (which you don't find often in aesthetic surgery), alcoholic liver cirrh. etc.*	*All **coagulation** abnormalities, V. Willebrand, V-Leiden mutation, etc.*	*The only useful risk assessment model for me currently is the Caprini Score for TVT*	***Procalcitonin***

	Wound healing complications, bleeding (experience from public hospital)	*Pause blood thinners, first optimize nutrition status, treat infection, if not related to the planed procedure.*		
Every of the above mentioned values is of concern if significantly out of range! normal lab values are to be checked by the GPs.				
Nutrition status, bleeding risk, infections are the main factors for me to decide about surgery.				
Only based on clinical exam and medical history				
*If a patient's **hemoglobin** level is low, I reject performing liposuction until the level improves, because during liposuction there is blood loss, which complicates the postoperative period.*				
*I am looking out for normal **coagulation parameters**. If these are out of range, I tend to be more cautious in my surgical approach and tend to choose less invasive options.*				*I think that **Procalcitonin**, especially in infected wounds, could be interesting.*
	*I have made the observation that under **albumin** substitution patients typically have shorter hospital stays.*	*When **platelets** are too low, i re-schedule the surgery if it's non-emergent.*	*Physiological status, as in preoperative lab values, should be taken more into account*	
As a hand surgeon, I like to understand what extent the inflammation has at the time of the surgery (f.i., rheumatic arthritis). Therefore, I like to order CRP to understand whether we have an inflammatory component to the arthrosis	*Subtle **coagulation** abnormalities or undiagnosed **thrombocytopenia** can be easily missed preoperatively. A full coagulation panel could have flagged a bleeding risk, especially in patients on anticoagulants or with liver disease.*		*Many current models are too generalized and don't reflect the nuances of different PRS procedures.*	
	Wound dehiscence, flap failure, hematoma formation, thromboembolic events, uncontrolled postoperative bleeding, and postoperative infections are some of the complications where thorough preoperative labs often provide early warning.	*Recently, a patient scheduled for an abdominoplasty was found to have **hypoalbuminemia** (albumin 2.8 g/dl). Surgery was postponed, and nutritional optimization was implemented. After normalization of albumin levels, the surgery proceeded uneventfully. In another case, elevated **INR** led to hematology consult and preoperative correction, likely preventing postoperative hematoma.*	*Specialty-specific algorithms that factor in wound healing biology, perfusion status, and soft tissue viability.*	***Prealbumin** as a more sensitive marker of short-term nutritional status compared to albumin.*<br>***Vitamin D levels***
	The unaddressed anemia, hyperglycemia, or clotting disorders are often linked to the development of wound infection, poor graft take, excessive bleeding, and delayed healing.	*Reduced surgical delay in transfusion and nutritional support was achieved by a low **hemoglobin** and **albumin** level which enhanced the viability of flaps and lowering the risk of postoperative risk.*	*Inclusion of nutritional status markers (**albumin, prealbumin, vitamin D**).*	***Albumin CRP** and **HbA1c** should be studied further to predict graft survival, wound healing period as well as the risk of infection in plastic surgery.*
*For example, **hypoalbuminemia** indicates poor nutritional status, which is predictive of wound healing complications. Elevated **creatinine** may reflect impaired renal function, impacting anesthesia risk and medication dosing. **Coagulation parameters (INR, PT, aPTT, platelets**) are critical for assessing bleeding risk, particularly in complex reconstructions or free flap procedures. Electrolytes like **potassium** and **sodium** affect cardiac stability perioperatively. **Blood glucose** and **CRP** are useful for identifying patients at risk for infections or delayed healing.*	*Poor **glucose** control in diabetics and low **albumin** leads to worse healing*	*I only perform elective surgery so i cancel any patients with medical problems.*	*Incorporation of machine learning to adapt risk models as more cases are added, rather than using outdated population data.*	*Nicotine and/or marijuana use*
The preoperative lab values will help determine the protocol and timing of surgery; the choice of a surgical technique; the reduction of risk; the reduction of comic-specific risks of infection, bleeding, wound healing and anesthesia clearance.	*Wound healing*		*Nutrition, inflammation and wound healing indices in plastic reconstructive surgery need to be better incorporated into risk models, particularly to cover burn, and trauma patients.*	***HbA1C***
	*Post weight loss surgery: **Pre-albumin***			
Inflammation parameters are something I have neglected for a long time. However, the immune response and the body's capabilities to cope with a major intervention such as surgery is weakened.			*I feel that the physiological status of each patient is insufficiently captured currently.*	*I feel that the physiological status of each patient is insufficiently captured currently.*
***Potassium** and **platelets** must be normal*		*Cancel for **platelets**, very high **glucose***	*Updated caprini scale*	***Prealbumin***

		Pt had unrecognized diabetes and surgery was cancelled.		
	None	*Factor V Leiden in a patient with a high caprini score and not previously evaluated.*		
For most patients considering major surgical procedures, they are referred to their own PCP, usually an internist, to undergo pre-operative evaluation and assessment, during which all of the above tests are performed. If any or some are out of the normal range, then significant consideration for postponement or even cancellation is given and discussed.				
	Infection in diabetic. Hematoma.	***Platelet** infusion in preop for known low platelets. Avoid breast surgery in known bleeding risk by **coags**. have patient's diabetes better managed before surgery.*		*I would like to have a recommended coworker beyond **PT/INR/PTT** for patients with history of excessive bleeding when these tests are normal.*
	None. history of dvt, and other items spark further evaluation	*Low **hemoglobin**, positive **pregnancy test**, **TSH**, **total protein**, **pre-albumin**, **drug testing** (amphetamine, cocaine, nicotine.*		*All the ones I mentioned. HCT, pregnancy, drug use. Then selected based on history, ETOH, heart, HTN, medications, weight loss. Not routine.*
*Only based on history. **hemoglobin** or **HCT** and **pregnancy test** and illicit **drug test** are used*				*Depending upon the case being performed a **pre-albumin** level is important (massive weight loss patients).*
	It is theoretically possible that a post-operative hematoma might have been predicted by someone with an abnormal leading time or PT/aPTT. But I rarely get serious post-operative complications and it makes no sense to test everyone for very infrequent genetic abnormalities	*When someone says they are diabetic, I've ordered **hemoglobin A1C**. If it is above 6.5, I do not operate on the patient until they have their sugars under better control. If someone is getting a breast reduction and they are an older patient, I will order a mammogram. If it is abnormal i will have them see a breast specialist prior to my performing surgery.*		
		*Elevated **WBC** before patient was diagnosed by their primary with a raging sinus infection lead us to delay a procedure. Positive **HCG** had lead us to cancel elective procedures.*		
*In some patients some of these values are useful. However in most of our health aesthetic patients, we only obtain an **HCG** level if they are less than two full years into menopause. In massive weight loss patients they must be stable at lowest weight for 18 months and have a good **albumin** level. In patients with history of anemia, **HCT** is useful. In patient with recent sinus infections (for example) or fairly recent illness we like to see a normal **WBC**. It varies patient to patient.*	*Delayed wound healing from unrecognized malnutrition*	*Anemia requiring transfusion*	*It is difficult to explain, but it is important discuss this topic, stimulate multicentric researches in order to offer objective guidelines to the practitioners and at the same time work in the patient education.*	
*Significant altered values can be related to disease condition as diabetes, anemia, inflammatory status. It is important include **homocystein** and **Vitamin D3** in the study.*				
	Wound healing problems	***Albumin** less than 3.5*		***Homocystein**, **Vitamin D3***
	Infections	*I will postpone surgery pending further consultation with patients personal physician and suggest a suggest an alternative solution if possible.*	*Always appreciate valid outcomes based studies on wound healing associated with affected patient types.*	***D-Dimer***
